# Tuning noise in gene expression

**DOI:** 10.15252/msb.20156210

**Published:** 2015-05-05

**Authors:** Sanjay Tyagi

**Affiliations:** Public Health Research Institute, Rutgers UniversityNewark, NJ, USA

## Abstract

The relative contribution of promoter architecture and the associated chromatin environment in regulating gene expression noise has remained elusive. In their recent work, Arkin, Schaffer and colleagues (Dey *et al*, 2015) show that mean expression and noise for a given promoter at different genomic loci are uncorrelated and influenced by the local chromatin environment.

See also: **SS Dey *et al*** (May 2015)

In eukaryotic organisms, many genes are expressed in episodic bursts that are interspersed with periods of quiescence (Raser & O'Shea, 2004; Chubb *et al*, 2006; Raj *et al*, 2006). Since the discovery of this phenomenon, scientists have wondered whether gene expression levels are controlled by the size or the frequency of these random bursts (Singh *et al*, 2010; Suter *et al*, 2011; Dar *et al*, 2012; Senecal *et al*, 2014).

mRNAs are usually short lived and begin to decay immediately after their synthesis. Therefore, the number of mRNA molecules present in a cell at a given moment depends on how long ago the burst of synthesis occurred and how large was the burst. Since the bursts of expression occur asynchronously, different cells have a different number of mRNA molecules at any given moment (Raj *et al*, 2006). This heterogeneity in gene expression is akin to electronic noise and is often analyzed as such.

**Figure 1:**
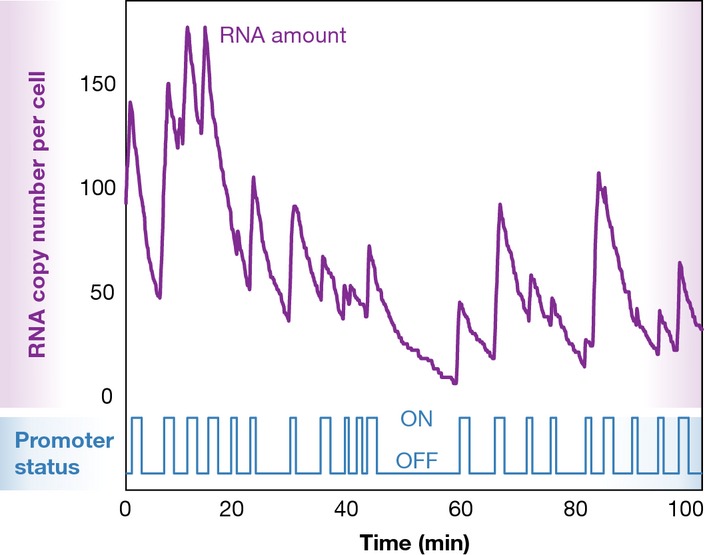
Bursts of mRNA synthesis are followed by steady decay of mRNA The figure shows a simulation of how the promoter status and RNA amounts change over time in a single cell under two-state model of transcription.

A plausible explanation for this pulsatile pattern of gene expression is that the regulatory regions of genes (promoters and enhancers) are usually sequestered within chromatin and become accessible to transcription factors (TFs) only intermittently (Raser & O'Shea, 2004; Raj *et al*, 2006). In addition to the state of local chromatin, gene expression levels also depend on the promoter architecture (which determines the strength of interaction between the promoter and its cognate TFs), the concentration of the TFs present in the cell and their ability to recruit the RNA polymerase complex to the gene locus. Since the architecture and accessibility of promoters both vary greatly across the genome, it is difficult to deconvolve the impact of these two factors on the characteristics of noise (Bar-Even *et al*, 2006). Dey *et al* (2015) focused on the contribution of the chromatin environment by integrating a reporter gene with the same promoter at many random locations within the genome and isolating 227 unique clones. For the construction of these clones, they exploited the twin abilities of the long terminal repeats (LTR) of HIV to direct the integration of its own genome at random locations within the human genome and to serve as a promoter for its coding sequences. However, instead of using the natural HIV genomic sequence, they used a GFP-coding sequence tagged with tandem sequence repeats in its 3′UTR. The former allowed them to quantify protein expression and the latter allowed them to measure mRNA expression with single-molecule sensitivity using fluorescent probes against the repeat.

By analyzing the distributions of GFP fluorescence among different cells of the clones, the authors show that integration at most genomic locations leads to pulsatile gene expression and individual clones exhibit very different mean levels of expression and noise (as measured by coefficient of variation (CV)). Protein reporters are less suited for examining the dynamics of gene activation events, because their steady state levels are buffered due to their relatively longer half-life. On the other hand, mRNAs, which are short lived and can be quantified by single-molecule fluorescence *in situ* hybridization (sm-FISH) are more suitable for this purpose (Raj *et al*, 2006). The authors’ sm-FISH analyses of representative pairs of clones that yield the same mean mRNA expression but different noise levels indicated that the burst size and burst frequency vary across genomic locations and they independently determine the mean and noise of gene expression. In particular, larger bursts drive the mean expression levels upward and higher burst frequencies turn the noise levels downward.

A surprising aspect of these results is that earlier studies that were also performed using the HIV LTR system had pointed out that both burst sizes and frequencies determine the mean expression levels (Singh *et al*, 2010; Dar *et al*, 2012). A possible explanation for the apparent discrepancy is that the particular technique used in the earlier studies (destabilized GFP) could have biased the analysis toward clones with higher expression, whereas the use of sm-FISH by Dey *et al* allows exploring a larger range of expression levels.

What differences in the local chromatin context can give rise to divergent noise in pairs of clones that show the same mean expression? Dey *et al* used DNase I sensitivity assays and examined the chromatin accessibility of the promoters in the different clones and found that the clone with higher noise in the pair always had less accessible chromatin, indicating that integration into more closed chromatin leads to noisier expression.

A limitation of DNAse I accessibility and chromatin immunoprecipitation techniques that are widely used to study the local chromatin environment is that they provide measurements of averaged ensembles of cells, whereas the phenomenon of transcriptional noise becomes apparent only at the single cell level. In this regard, single-molecule nucleosome occupancy analysis by electron microscopy in yeast has recently emerged as an exciting new development (Brown & Boeger, 2014). This analysis has revealed that promoters stochastically assume nucleosome-free and nucleosome-occupied states that likely correspond to the expressive and quiescent states of genes.

The study of Dey *et al* raises several interesting questions. Since *a priori* increases in both the burst size and the burst frequency are expected to lead to increased expression, why do cells use only burst size to accomplish higher expression? What kind of molecular mechanisms exist in the cell that allow for independent controls of burst size and burst frequency? And finally, how do the promoter architectures and the surrounding chromatin interact to determine accessibility to TF and noise characteristics? Future studies addressing these questions are expected to further advance our understanding of the molecular and epigenetic mechanisms underlying the control of gene expression noise and the resulting phenotypic heterogeneity.
